# One-Dimensional Mn_5_Si_3_ Nanorods: Fabrication, Microstructure, and Magnetic Properties via a Novel Casting-Extraction Route

**DOI:** 10.3390/ma16093540

**Published:** 2023-05-05

**Authors:** Hang Li, Dongtao Niu, Zhongtao Zhang, Fan Yang, Hongxia Wang, Weili Cheng

**Affiliations:** 1 School of Material Science and Engineering, Taiyuan University of Technology, Taiyuan 030024, China; 2 Golden Dragon Precise Copper Tube Group Inc., Chongqing 404100, China

**Keywords:** manganese silicide, nanorods, microstructure, ferromagnetic

## Abstract

This study presents a simple and innovative approach for producing one-dimensional Mn_5_Si_3_ nanorods through a casting-extraction process. In this technique, the Mn_5_Si_3_ nanorods were synthesized by reacting Mn and Si during brass solidification and extracted by electrochemical etching of the brass matrix. The effect of the cooling rate during casting on the nanorods’ dimension, morphology, and magnetic properties was investigated. The results demonstrate that the prepared high-purity Mn_5_Si_3_ nanorods had a single-crystal D8_8_ structure and exhibited ferromagnetism at room temperature. The morphology of the nanorods was an elongated hexagonal prism, and their preferred growth was along the [0001] crystal direction. Increasing the cooling rate from 5 K/s to 50 K/s lead to a decrease in the dimension of the nanorods but an increase in their ferromagnetism. At the optimal cooling rate of 50 K/s, the nanorods had a diameter and length range of approximately 560 nm and 2~11 μm, respectively, with a highest saturation magnetization of 7.5 emu/g, and a maximum coercivity of 120 Oe. These properties make the fabricated Mn_5_Si_3_ nanorods potentially useful for magnetic storage applications, and this study also provides a new perspective on the preparation of one-dimensional nanomaterials.

## 1. Introduction

One-dimensional (1D) nanomaterials (nanowires, nanorods, nanobelts, etc.) are a research focus in the scientific community due to their unique structures and integration of unusual physical properties. With unique advantages in the fields of electrics, optics, and magnetism, 1D nanomaterials hold immense promise in relevant nanotechnology applications [1,2,3,4,5,6,7,8,9,10]. Recently, nanostructured manganese silicides, including MnSi, Mn_5_Si_3_, Mn_3_Si, etc., received significant attention for their potential use in magnetic and spintronic applications [11,12,13,14,15,16]. Among these silicides, Mn_5_Si_3_, synthesized in the form of nanoparticles, nanowires or nanorods, was shown to exhibit impressive magnetic properties, including high magnetic moment, saturation magnetization and coercivity [12,15,17,18]. As a result, Mn_5_Si_3_ shows particular promise for use in magnetic storage devices. Its hexagonal structure and spin textures make it a promising candidate for creating high magneto-crystalline anisotropy. Additionally, nanocrystal Mn_5_Si_3_ has a drastically improved magnetic ordering temperature due to the size effect and can be ferromagnetic at room temperature, opening up new possibilities for practical applications [19,20].

Various methods were attempted to fabricate nanostructured Mn_5_Si_3_, including chemical vapor deposition (CVD), sputtering and laser deposition. For example, Higgins et al. [21] synthesized the Mn_5_Si_3_ nanowires by CVD through the direct reaction of Mn vapor with a Si substrate. Hamzan et al. [17] improved the growth of Mn_5_Si_3_ nanorods on a Si/SiO_2_ substrate using CVD by increasing the reaction temperature. Lu et al. [18] reported a solid-state route for preparing Mn_5_Si_3_ nanorods using Mn_2_O_3_, Si and Mg as reactants. In addition, Das et al. [15] fabricated the Mn_5_Si_3_ nanoparticles through direct current magnetron sputtering. Rylkov et al. [22] produced nano-thick Mn_5_Si_3_ films using pulsed laser deposition. However, these methods have some limitations, such as the need for complex and expensive equipment, harsh reaction conditions (such as high temperatures or pressures), and the formation of unwanted by-products, including other Mn-Si compounds and oxides [23,24]. These limitations restrict the large-scale preparation of high-quality Mn_5_Si_3_ nanomaterials.

Recently, Wang et al. [25] proposed a novel strategy for the preparation of Ti_5_Si_3_ nanowires through a casting-extraction method. In this process, Ti_5_Si_3_ nanowires were synthesized in a brass melt and were then extracted by electrochemical corrosion of the brass matrix. This method offers several advantages, such as its simple device and process, low cost, and ease of large-scale production. The resulting defect-free Ti_5_Si_3_ nanowires are high-purity, single-crystal materials and exhibit good electrical properties. The formation of Ti_5_Si_3_ nanowires takes advantage of its low solubility in the Cu-Zn alloy and the preferential growth direction of the D8_8_ hexagonal structure silicides. It is worth noting that this casting-extraction method is only suitable for a few specific nanomaterials. Nevertheless, it is possible to extend the method to the preparation of 1D Mn_5_Si_3_ nanomaterials, given the similarities in solubility in Cu-Zn alloy, crystal structure and growth pattern between Ti_5_Si_3_ and Mn_5_Si_3_ [26,27,28].

In the present study, we successfully fabricated Mn_5_Si_3_ nanorods by combining the casting of brass containing Mn and Si, and the extraction through an electrochemical dissolution of the brass matrix. The effect of the cooling rate during the casting process on the structure, growth, morphology, magnetic properties, and oxidation resistance of the as-prepared Mn_5_Si_3_ nanorods was investigated. Our findings may provide valuable insights into the fabrication of silicide nanomaterials, which could have various potential applications.

## 2. Experimental Method

### 2.1. Preparation Process of Mn_5_Si_3_ Nanorods

**Figure 1:**
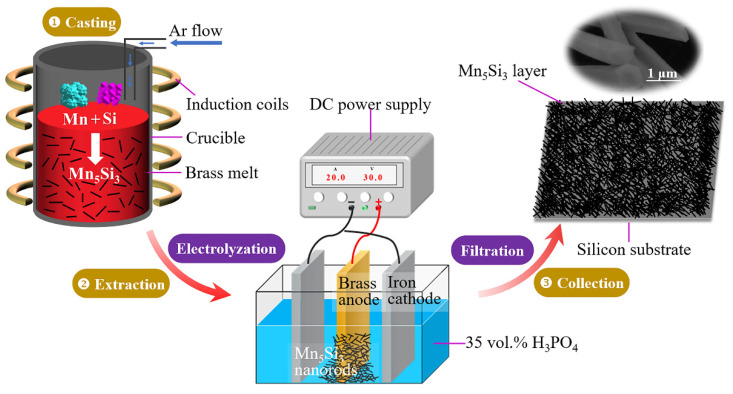
Schematic diagram showing the preparation process of Mn_5_Si_3_ nanorods.

Figure 1 illustrates the entire process for preparing Mn_5_Si_3_ nanorods, which consisted of three steps. The first step was casting a brass slab containing Mn and Si. The pure Si (99.9%), Cu-30 wt% Mn and Cu-35wt% Zn-3wt% Al master alloys were melted in a graphite crucible using a medium-frequency induction furnace, protected by high-purity argon gas. The melt was held at 1373 K for 5 min and then poured into a steel or copper mold. The resulting brass slab had dimensions of 100 mm × 100 mm × 10 mm. The addition of Mn and Si in a 5:3 molar ratio aimed to produce Mn_5_Si_3_ nanorods. This compound formed as a result of a chemical reaction between Mn and Si, which released energy in the form of heat. The reaction equation for this process was 5Mn + 3Si → Mn_5_Si_3_ + ΔH_f_ = −200.9 kJ/mol, where ΔH_f_ was the enthalpy change for the reaction [29]. The solid solution of Al served to form a β matrix (Cu-rich, bcc crystal structure) in the brass, which was less corrosion-resistant than other types of matrix structure, favoring rapid corrosion of the brass matrix [30]. The second step was the electrochemical extraction of Mn_5_Si_3_ nanorods. The brass matrix as anode was corroded rapidly by using a phosphoric acid solution (35 vol.% H_3_PO_4_) as an electrolyte with applying a direct current (DC), leaving behind the Mn_5_Si_3_ nanorods. The nanorods were then separated from the solution through several filtration passes. In step three, the collected Mn_5_Si_3_ nanorods were poured into anhydrous ethanol, ultrasonically cleaned and then dropped onto a small Si substrate (1 cm × 2 cm). The Mn_5_Si_3_ layer was obtained after natural air drying.

Brass alloys with different Mn and Si compositions were cast to investigate the potential formation of Mn_5_Si_3_ nanorods. Table 1 shows the nominal compositions of three experimental brass alloys. In addition, to create varied cooling rates during the casting process, a steel mold, a copper mold, and a copper mold equipped with a circulating water-cooling device were used, resulting in cooling rates of approximately 5 K/s, 25 K/s, and 50 K/s, respectively.

### 2.2. Characterization of Mn_5_Si_3_ Nanorods

The phase composition of the brass alloys and the crystal structure of the prepared nanorod samples were analyzed by an X-ray diffractometer (XRD, Rigaku Ultima IV, Tokyo, Japan). The purity of the Mn_5_Si_3_ nanorods was determined through X-ray fluorescence (XRF, Shimadzu XRF1800, Kyoto, Japan). The yield was calculated from the ratio of acquisition to addition using an analytical balance with a precision of 0.0001 g. The microstructures were characterized using a scanning electron microscope (SEM, Tescan Mira 3XMU, Brno, Czech Republic) equipped with an energy dispersive spectrometer (EDS), and a transmission electron microscope (TEM, JEOL JEM-F200, Tokyo, Japan). Magnetic properties were measured using a vibrating sample magnetometer (VSM, LakeShore-7404, Westerville, OH, USA) with a maximum applied magnetic field of 10 KOe at room temperature. Thermogravimetric (TG) analysis and differential thermal analysis (DTA) were carried out using a thermoanalyser apparatus (Mettler-Toledo, TGA/SDTA851, Columbus, OH, USA) to evaluate the oxidation resistance of Mn_5_Si_3_ nanorods in air. The nanorod samples were heated from room temperature to 1273 K at a constant rate of 10 K/min.

## 3. Results and Discussion

### 3.1. Microstructure Characterization

**Figure 2:**
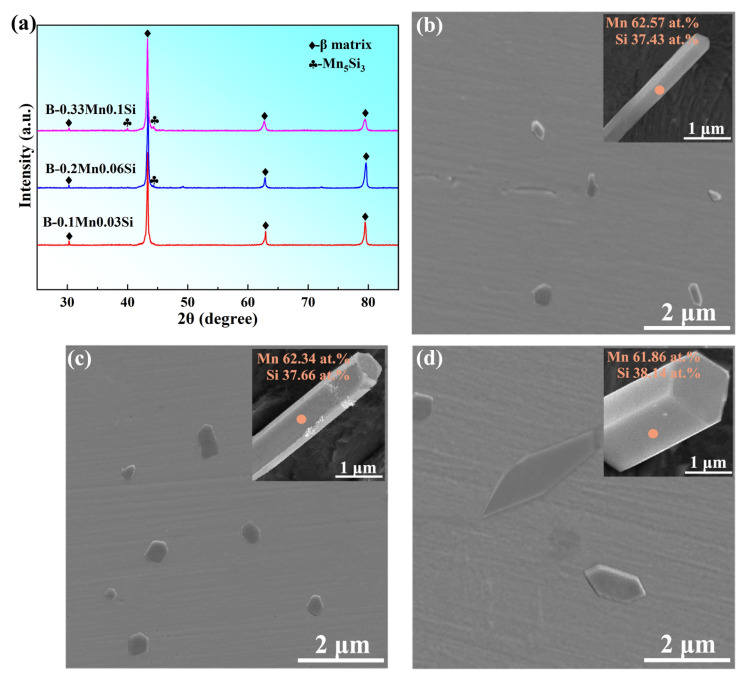
(**a**) XRD patterns and SEM images of different as-cast brass alloys: (**b**) B-0.1Mn-0.03Si; (**c**) B-0.2Mn-0.06Si and (**d**) B-0.33Mn-0.1Si; insets show the 3D observation and EDS analysis of the Mn_5_Si_3_ phase in brasses after deep etching.

Figure 2a shows the XRD patterns of three brass alloys with different Mn and Si compositions. It is apparent that the brasses consisted mainly of β phase. The diffraction peaks of the Mn_5_Si_3_ phase were not clearly visible due to the small content in the alloys. The corresponding microstructures of the brasses are shown in Figure 2b–d. The grey matrix represents the β phase and the particles represent the Mn_5_Si_3_ phase. After conducting a deep etching process on the brass matrix, the 3D morphology observation with EDS analysis revealed that the Mn_5_Si_3_ particles had a long, hexagonal prism shape. In the B-0.1Mn-0.03 Si alloy with lower Mn and Si contents, the generated Mn_5_Si_3_ particles were nano-sized in diameter and small in number (Figure 2b). The number of particles increased with increasing Mn and Si contents in the B-0.2Mn-0.06Si alloy (Figure 2c). As the Mn and Si contents were further increased, the dimension of the Mn_5_Si_3_ phase increased and the diameter of most particles exceeded the micron level in the B-0.33Mn-0.1Si alloy (Figure 2d). Based on these observations, the B-0.2Mn-0.06Si alloy with a suitable number and size of particles was considered the best option for the preparation of Mn_5_Si_3_ nanorods through the casting-extraction method. Unless otherwise stated, the as-cast B-0.2Mn-0.06Si slab was used in the subsequent work of this study.

**Figure 3:**
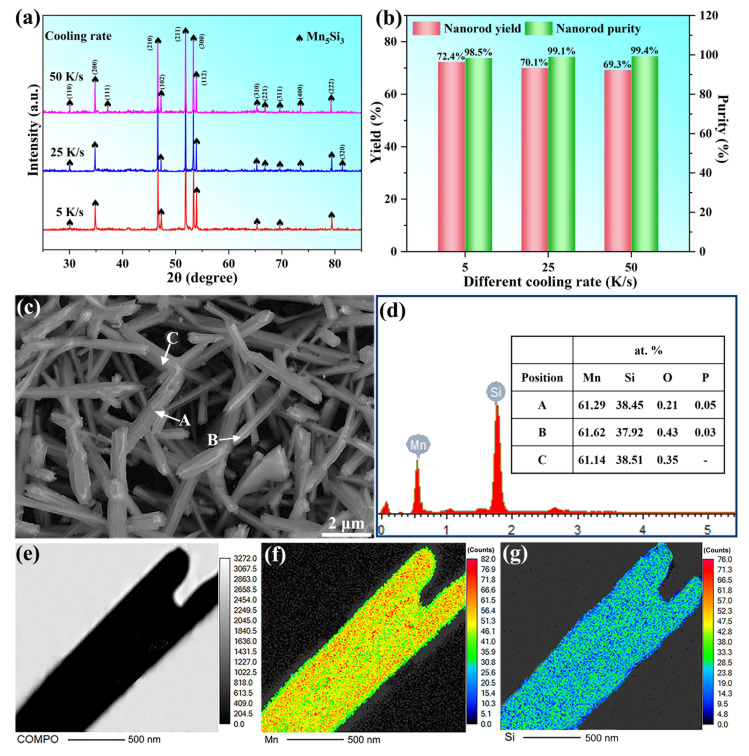
Different test results of collected Mn_5_Si_3_ nanorods: (**a**) XRD patterns; (**b**) yield and purity; (**c**) SEM observation; (**d**) corresponding EDS analysis for the spots A, B and C on different nanorods in (**c**); EDS mapping analysis showing the element distribution, (**e**) BSE image; (**f**) Mn element; (**g**) Si element.

Figure 3a displays the XRD patterns of the collected nanorod samples prepared under casting conditions with different cooling rates. It demonstrates that the prepared nanorod was single-crystal Mn_5_Si_3_, while no other manganese silicides were detected. Figure 3b shows the yield and purity results for Mn_5_Si_3_ nanorods. The yield remained consistently high at around 70% under all three conditions. The yield loss may have been due to the failure of some nanorods to grow, which were removed during several rounds of filtration. As the cooling rate increased from 5 K/s to 50 K/s, the yield decreased slightly from 72% to 69%. This was because the higher cooling rate lead to a shorter growing time of Mn_5_Si_3_ during solidification and promoted the formation of non-growing particles, which can be more easily filtered out. Moreover, the purity of the obtained Mn_5_Si_3_ nanorods prepared under the three conditions was all greater than 98.5%, indicating a low impurity content. In Figure 3c, the SEM image illustrated Mn_5_Si_3_ nanorods prepared at a 5 K/s cooling rate during casting, with a significant number of nanorods ranging from 4 to 16 μm in length. Figure 3d shows the results of EDS component analysis on different nanorods, revealing that the Mn_5_Si_3_ nanorods were primarily composed of Mn and Si, with only a trace amount of impurities (O and P) introduced during the electrolytic dissolution of the brass matrix. Therefore, the cast-extraction method was proven to be an effective way to prepare pure, single-crystal Mn_5_Si_3_ nanorods with high yields. Figure 3e–g demonstrate the EDS mapping results of the Mn_5_Si_3_ nanorod, indicating that both Mn and Si elements were uniformly distributed throughout the entire nanorod length.

**Figure 4:**
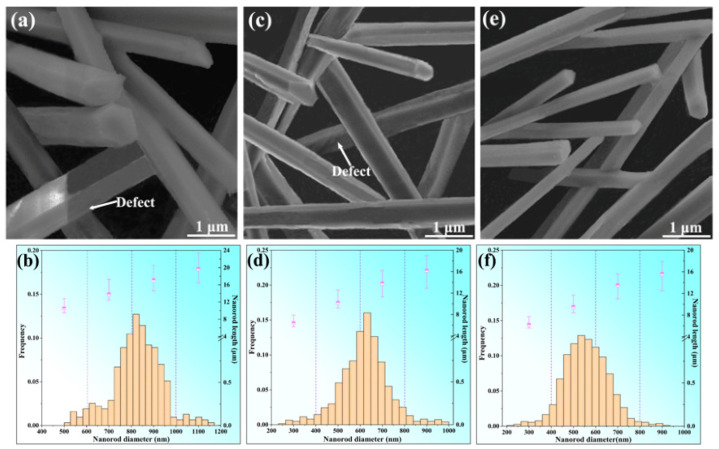
SEM images and dimension distribution of Mn_5_Si_3_ nanorods prepared with different cooling rates during casting: (**a**,**b**) 5 K/s; (**c**,**d**) 25 K/s; (**e**,**f**) 50 K/s.

Figure 4a–c shows the typical 3D morphology of Mn_5_Si_3_ nanorods prepared under casting conditions with cooling rates of 5~50 K/s. In all cases, the nanorods exhibited the elongated hexagonal prism morphology, which was consistent with the observation in the brass microstructure. Additionally, the nanorods exhibited a uniform diameter along their entire lengths, and the dimension decreased as the cooling rate increased. At the cooling condition of 5 K/s and 25 K/s, only a few small defects were observed on the prism surfaces of the nanorods. However, at the cooling rate of 50 K/s, the defects on the nanorod surface were hardly noticeable, indicating relatively complete crystal growth. Statistical analysis was conducted on hundreds of as-prepared Mn_5_Si_3_ nanorods for each casting condition, and their size distributions are shown in Figure 4d–f. When the cooling rate was 5 K/s, 25 K/s, and 50 K/s, the average diameter of the nanorods was 850 nm, 680 nm, and 560 nm, and the length range was 4~16 um, 3~12 um, and 2~11 um, respectively. For most nanorods, it was found that there was an almost linear relationship between the length and diameter, and the aspect ratio of the nanorods exceeded 10 and remains relatively constant despite changes in the cooling rate. The dimension and morphology of the as-prepared nanorods depend on the formation and crystal growth of Mn_5_Si_3_ during the brass solidification. Previous studies on manganese silicon brasses showed that Mn_5_Si_3_ formed the primary phase and grows in the brass melt [27]. As the cooling rate of the melt increased from 5 K/s to 50 K/s, the growth of Mn_5_Si_3_ was inhibited, leading to a decrease in the nanorod dimension. The hexagonal prism morphology of the Mn_5_Si_3_ nanorods observed in this study was in good agreement with previous studies. It was widely believed that the hexagonal prism growth of Mn_5_Si_3_ is closely related to its crystal structure [26,31,32,33]. Moreover, the long rod-like shape of the crystals is typically formed by rapid growth in a preferred orientation due to its structural anisotropy [34]. Therefore, the crystal structure of Mn_5_Si_3_ may significantly influence its growth morphology, which will be analyzed in detail later in this article.

**Figure 5:**
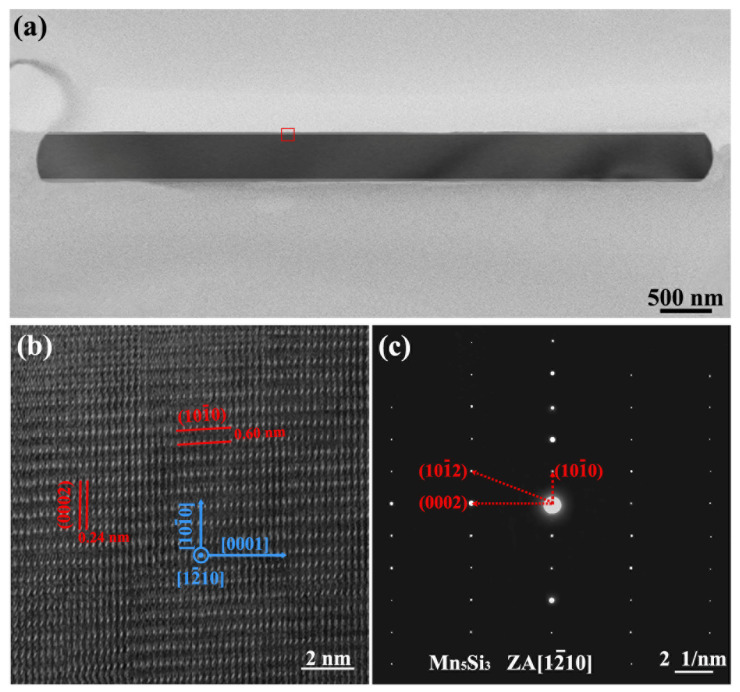
TEM analysis of the Mn_5_Si_3_ nanorod prepared at a cooling rate of 50 K/s during casting: (**a**) bright field image; (**b**) HRTEM image of the nanorod edge shown in marked area in (**a**); (**c**) SAED pattern along the [1$\bar{2}$10] zone axis.

To learn more about the crystal structure of Mn_5_Si_3_ nanorods, the TEM examination was carried out on the nanorods prepared at 50 K/s cooling rate during casting. Figure 5a shows the TEM bright field image of the Mn_5_Si_3_ nanorod. The high-resolution TEM (HRTEM) image of the nanorod edge (the marked area in Figure 5a) is displayed in Figure 5b. It can be found that the measured interplanar distances in the two orthogonal directions were 0.60 nm and 0.24 nm, which corresponded to the *d*-spacings of the (0002) and (10$\bar{1}$0) crystal faces, respectively. It indicates that the prism height, in other words the growth direction of the nanorods, was parallel to the [0001] direction, while the prism side planes were (10$\bar{1}$0) faces. The selected area electron diffraction (SAED) pattern (Figure 5c) further confirmed that the Mn_5_Si_3_ nanorod had a single crystal phase with the D8_8_ hexagonal structure. The calibration results of the diffraction patterns were consistent with the HRTEM image observation.

### 3.2. Formation and Growth Mechanism of Mn_5_Si_3_ Nanorods

**Figure 6:**
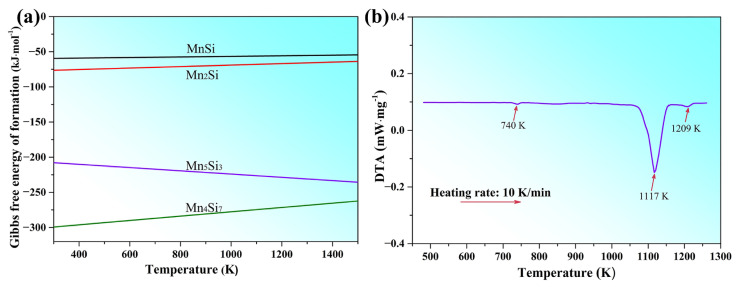
(**a**) Gibbs free energies of formation with respect to temperature for the manganese silicides and (**b**) DTA curve of the B-0.2Mn-0.06Si alloy.

This study revealed that only Mn_5_Si_3_ was detected in the as-prepared nanorods, indicating the formation of a single compound phase in the as-cast B-0.2Mn-0.06Si alloy. To comprehend the absence of various manganese silicides in the alloy system, Figure 6a illustrates the Gibbs free energies of formation for potential silicides as a function of temperature. The values at four temperatures were taken from ref. [29], while the other values were obtained by linear interpolation. This shows that Mn_5_Si_3_ and Mn_4_Si_7_ have much lower formation energies than Mn_2_Si and MnSi, particularly at higher temperatures, suggesting their higher thermodynamic stability and stronger formation tendency. The presence of Mn and Si in a 5:3 molar ratio in the B-0.2Mn-0.06Si alloy further resulted in the preferential formation of the Mn_5_Si_3_ phase during solidification. In addition, DTA analysis was carried out on this alloy to determine the temperature range at which Mn_5_Si_3_ forms, as illustrated in Figure 6b. During the heating process, the DTA curve exhibited three endothermic peaks. The first peak, a small one at around 740 K, was caused by the phase transition from disordered β’ to ordered β, which is common in brass alloys. The second, large peak at around 1117 K, was produced by the melting of the β matrix. The third peak around 1209 K, corresponds to the dissolution of the Mn_5_Si_3_ phase in the brass melt. Hence, during solidification of the B-0.2Mn-0.06Si alloy, Mn_5_Si_3_ formed as the primary phase in the high-temperature melt, followed by the crystallization of the β phase.

**Figure 7:**
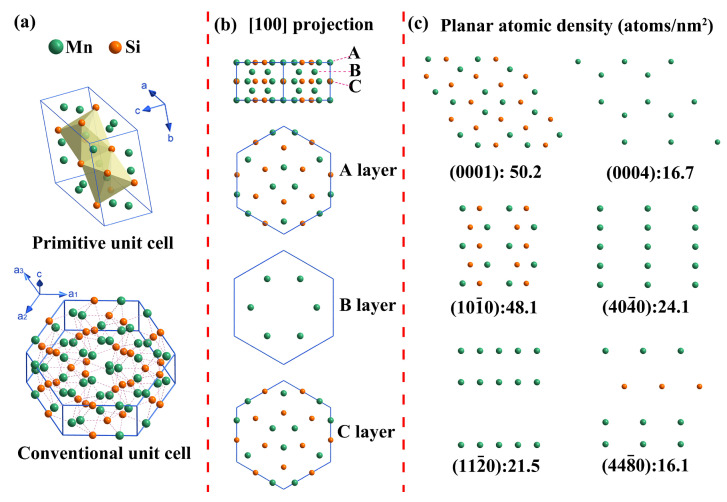
Crystal structure of Mn_5_Si_3_: (**a**) primitive and conventional unit cells; (**b**) side view of unit cell and atomic distributions of layers A, B, and C; (**c**) atomic distribution with reticular density of different crystal faces.

Since the crystal structure is the internal factor determining the growth morphology of Mn_5_Si_3_ during solidification, it was in-depth analyzed to better understand the growth mechanism of Mn_5_Si_3_ nanorods. Mn_5_Si_3_ has a D8_8_-type hexagonal structure with the space group of P6_3_/mcm and the lattice constants of a = 0.691 nm and c = 0.481 nm [35]. Figure 7a shows the primitive and conventional unit cells of the Mn_5_Si_3_, respectively. The latter contained 10 Mn atoms and 6 Si atoms, with the Mn atoms at the equivalent point positions 4d (0.33, 0.67, 0) and 6g (0.23, 0, 0.25) and the Si atoms at the equivalent point position 6g (0.60, 0, 0.25). A projection in the [100] direction showed that the crystal unit cell had five atomic layers along the *c*-axis direction, with an ‘ABCBA’ stacking order (Figure 7b). The atomic arrangement of the layers A and C was the same but rotated by 180 degrees. Figure 7c presents the atomic distribution with reticular density for different crystal faces. The (0001) and (10$\bar{1}$0) faces were found to be close-packed with higher reticular densities. The crystal structure of Mn_5_Si_3_ showed complex symmetry and pronounced anisotropy, which promotes varied growth rates along different crystal directions. According to classical growth theory, crystals with lattice constants a ≈ b ≈ c typically grow into symmetrical shapes, such as a cube, tetrahedron, and octahedron. Mn_5_Si_3_ belongs to the class of crystals with lattice constants a ≈ b > c, which generally grow into a prismatic shape [34]. Thus, during the growth of Mn_5_Si_3_, the preferred growth direction is <0001>, resulting in the growth order of the crystal faces being (0001) → (0004) → (0002). In the plane perpendicular to the <0001> direction, the (11$\bar{2}$0) face with lower atomic density grew at a faster rate and was more likely to disappear. In contrast, the close-packed (10$\bar{1}$0) face grows at a lower rate and was more likely to be exposed. According to the Bravais-Friedel law, the close-packed (0001) and (10$\bar{1}$0) faces with lower surface energy tend to be preserved after the eventual crystal growth [36]. Given that the Mn_5_Si_3_ has a higher melting entropy and presents a typically faceted growth pattern, it tends to form a long, hexagonal prism morphology. The prism basal plane is referred to as the (0001) face with the prism side plane the (10$\bar{1}$0) face, which is consistent with the TEM results. Additionally, the solute concentration of Mn and Si in the B-0.2Mn-0.06Si alloy melt was lower, which restricts the diameter of Mn_5_Si_3_ prism to only grow to nanometer size.

Increasing the cooling rate from 5 K/s to 50 K/s did not alter the faceted growth pattern or the growth rate ratio of dominant growth direction <0001> to <11$\bar{2}$0>. Thus, Mn_5_Si_3_ still grew into a long, hexagonal prism with small changes in aspect ratio. However, it was inevitable to prevent crystal defects from forming during the growth of Mn_5_Si_3_ due to the limited solute attachment kinetics. Maintaining the growth of flat facets requires solute atoms to continuously adsorb onto lattice sites on the prism surface [37]. As the crystal grew, the area of the prism side planes increased rapidly while the solute concentration in the surrounding melt decreased. Defects will form in localized regions of the prism side plane when solute adsorption cannot sustain faceted growth. Therefore, increasing the cooling rate from 5 K/s to 50 K/s lead to a reduction in the dimension of Mn_5_Si_3_ nanorods with a decreased defect size.

### 3.3. Magnetic Properties

**Figure 8:**
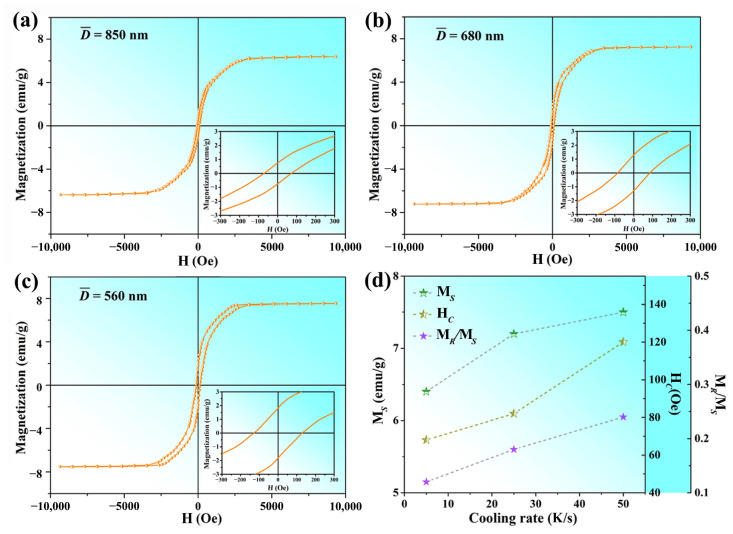
Magnetic hysteresis loops of the Mn_5_Si_3_ nanorods prepared with different cooling rates during casting: (**a**) 5 K/s; (**b**) 25 K/s; (**c**) 50 K/s; (**d**) variations of the M*_S_*, H*_C_*, and M*_R_*/*M_S_* with respect to the cooling rate.

The magnetic properties of nanorod samples prepared under different casting conditions were evaluated at room temperature. A magnetic field ranging from −10 kOe to 10 kOe was applied and the resulting hysteresis loops are depicted in Figure 8a–c. It can be observed that all the Mn_5_Si_3_ nanorods exhibited ferromagnetic behavior. The saturation magnetization (M*_S_*), coercivity field (H*_C_*), and remanence-to-saturation magnetization ratio (M*_R_*/M*_S_*) were analyzed statistically, and their dependence on cooling rate during casting is demonstrated in Figure 8d. The curves show that M*_S_*, H*_C_*, and M*_R_*/M*_S_* increased gradually as the cooling rate increased from 5 k/s to 50 k/s. Specifically, at a cooling rate of 5 K/s, M*_S_* and H*_C_* were measured to be 6.4 emu/g and 71 Oe, respectively. As the cooling rate increased to 25 K/s, M*_S_* and H*_C_* increased to 7.1 emu/g and 80 Oe, and reach to maximum values of 7.5 emu/g and 120 Oe when the cooling rate reached 50 K/s. The M*_R_*/M*_S_* value also increased from 0.11 to 0.24 over the same cooling rate range. These results suggest that Mn_5_Si_3_ nanorods prepared under 50 K/s cooling condition during casting exhibited optimal magnetic properties. Table 2 summaries the dimension and magnetic properties of nanostructured Mn_5_Si_3_ prepared via different methods in previous literature. The M*_S_* and H*_C_* of the nanorods prepared under the optimal casting condition in this work were superior to those reported by Lu et al. [18] and Hamzan et al. [17] using the CVD method. Although the reasons for this phenomenon are unclear, it is tentatively proposed that the achievement of pure, single-crystal Mn_5_Si_3_ nanorods plays a crucial role, since the formation of other byproducts was reported in both CVD methods. However, our nanorods exhibited lower magnetic properties compared to the Mn_5_Si_3_ nanoparticles fabricated by Das et al. using the magnetron sputtering method. Furthermore, the M*_S_* value remained inferior to that of certain well-developed magnetic storage materials, such as bulk γ-Fe_2_O_3_ (76 emu/g) [38], bulk CoFe_2_O_4_ (80 emu/g) [39], and Fe_3_O_4_ nanoparticles (45 emu/g) [40]. The reasons behind this result are multifaceted, and one possible explanation relates to the crystal structure of the materials. Mn_5_Si_3_ has a weak magnetic moment compared to γ-Fe_2_O_3_ and CoFe_2_O_4_, which have stronger magnetic interactions, as well as Fe_3_O_4_ with a higher magneto-crystalline anisotropy. In addition, our nanorods had diameters of several hundred nanometers and, thereby, exhibited less prominent size effects. Nevertheless, the prepared Mn_5_Si_3_ nanorods hold great potential for further optimization to maximize magnetization.

Bulk Mn_5_Si_3_ is paramagnetic at room temperature, whereas the nano-sized Mn_5_Si_3_ produced in this research demonstrated enhanced magnetization. It implied a possible ferromagnetic ordering with a Curie temperature higher than 300 K. It is widely recognized that the magnetic properties of nanomaterials were determined by a complex interplay of factors, including their structural composition, dimensions, morphologies, surface disorder, and the presence of any defects or impurities [41,42]. In the case of one-dimensional nanomaterials, shape anisotropy significantly affects their saturation magnetization and coercivity [43]. The shape anisotropy of nanorods can heighten the probability of magnetic moments aligning along the long axis due to the magneto-crystalline anisotropy. The strength of the shape anisotropy effect depends on the nanorod aspect ratio. As the aspect ratio increased, the shape anisotropy effect became stronger, leading to increased magnetization. However, this also made it more challenging to switch the magnetic moment to align with an external magnetic field, which ultimately lead to an increase in coercivity. Therefore, the Mn_5_Si_3_ nanorods prepared by this casting-extraction method exhibited a single-crystalline D8_8_ structure and a high aspect ratio, thereby displaying ferromagnetism with appreciable saturation magnetization and coercivity.

As the cooling rate during casting increases from the 5 K/s to 50 K/s, the enhancement in magnetic properties can be attributed mainly to the reduction in the size of the Mn_5_Si_3_ nanorods, since all nanorods produced under different casting conditions have the same crystal structure and prism-like morphology with no apparent defects. In terms of saturation magnetization, the nanoscale effect becomes more pronounced as the average diameter of the nanorods decreases from 850 nm to 560 nm. Meanwhile, the surface/volume ratio of the nanorods increases with a higher surface effect. According to the study by Das et al. [15], the surface atoms of nanostructured Mn_5_Si_3_ have large spin polarization and high magnetic moments. Therefore, the enhanced surface effect can lead to an increase in the saturation magnetization of Mn_5_Si_3_ nanorods. Concerning coercivity, the magnetic moments of smaller nanorods are more susceptible to thermal fluctuations and external magnetic fields, resulting in a higher coercivity [44]. The surface effect that arises with decreasing nanorod diameter can also contribute to an increase in coercivity. This is because the surface atoms on the nanorods can undergo rearrangement, resulting in the reversal of magnetic anisotropy [45]. In addition, the M*_R_*/M*_S_* ratio also increases as the nanorod size decreases. This trend is attributed to the increase in magnetic anisotropy energy due to the surface effect [46]. The nanorod is more likely to retain its magnetic moment in the absence of an external magnetic field, which contributes to a higher remanence.

### 3.4. Oxidation Resistance

**Figure 9:**
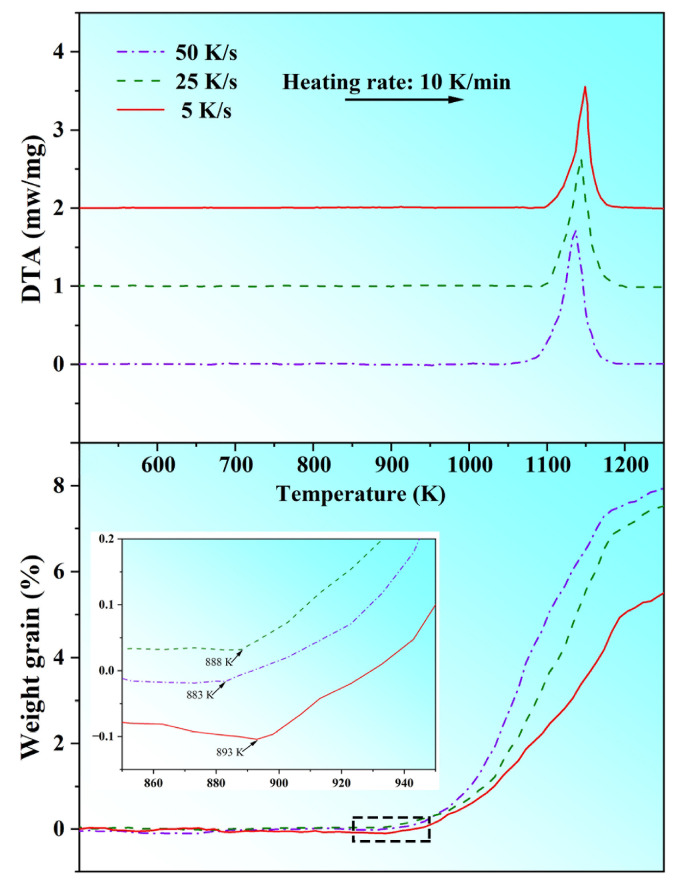
TG/DTA curves of different nanorod samples prepared with different cooling rates during casting, inset showing the onset oxidation temperatures of Mn_5_Si_3_ nanorods.

To investigate the oxidation resistance of the Mn_5_Si_3_ nanorods in this study, various samples prepared under different casting conditions were analyzed by TG-DTA from room temperature to 1273 K in air and the results are depicted in Figure 9. The TG curves revealed that the oxidation process of all samples followed a similar pattern concerning temperature changes. Upon reaching the oxidation onset temperature, the initial weight gain due to oxidation occurred rapidly, while the oxidation process slowed down as the temperature rose. This behavior can be attributed to the formation of a protective oxide layer. By examining the TG curves at a local magnification, it was observed that the onset temperatures of oxidation reaction for samples with cooling rates of 5 K/s, 25 K/s, and 50 K/s during casting were approximately 893 K, 888 K, and 883 K, respectively. As the cooling rate increased, the oxidation onset temperature gradually decreased while the weight gain increased, indicating a decline in the oxidation resistance of the Mn_5_Si_3_ nanorods. This was likely due to the smaller nanorod size resulting in a larger specific surface area, since several studies showed that nanomaterials with greater surface area are more susceptible to oxidation [47,48]. Nonetheless, the weight gain of all samples remains below 8%, demonstrating that, overall, the Mn_5_Si_3_ nanorods produced in this study had good oxidation resistance. Based on the DTA curves, it was evident that all samples displayed a significant exothermic peak around 1150 K, indicating that the oxidation reaction of Mn_5_Si_3_ nanorods was endothermic.

### 3.5. Prospects and Advancements for Applications

The current study utilized a novel casting-extraction method to fabricate Mn_5_Si_3_ nanorods, which only requires conventional casting and electrolysis devices, and simple and cost-effective technological processes. The as-prepared Mn_5_Si_3_ nanorods exhibited high quality with a good yield and appreciable ferromagnetic properties at room temperature. As a result, the casting-extraction method holds great potential for the large-scale production of Mn_5_Si_3_ nanorods applied for magnetic storage devices. However, since this study was exploratory, there is scope for advancements in many aspects of the experimental process, particularly concerning large-scale production. For instance, large-sized manganese-silicon brass ingots can be prepared at once and used for multiple electrolytic extractions, thus eliminating one process step and reducing costs. Centrifugal filtration can replace conventional atmospheric pressure filtration to separate nanorods and electrolyte, minimizing the loss of nanorods during the transfer process following extraction. In addition, rapid solidification or adding modifying elements might be introduced into the brass casting to control the size and morphology of Mn_5_Si_3_ nanorods, thereby further improving their magnetic properties [27,32]. Moreover, given the identical crystal structure and similar thermodynamic properties of D8_8_-type silicides, the casting-extraction method also presents a promising approach for fabricating 1D nanomaterials of silicides other than Ti_5_Si_3_ and Mn_5_Si_3_, such as Mo_5_Si_3_, Fe_5_Si_3_, Cr_5_Si_3_, etc., which hold immense potential for diverse nanotechnological applications [49,50,51].

## 4. Conclusions

Via a simple and innovative casting-extraction method, our study successfully produced Mn_5_Si_3_ nanorods with a high purity exceeding 98.5% and a good yield of about 70%. The effect of the cooling rate during casting on the nanorods’ dimension, morphology, and magnetic properties was investigated. The results indicate that the as-prepared Mn_5_Si_3_ nanorods exhibited a single-crystal D8_8_ structure, elongated hexagonal prism morphology with no apparent defects, and preferential growth along the [0001] crystal direction. Increasing the cooling rate from 5 K/s to 50 K/s reduced the dimensions of the nanorods but increased the ferromagnetism at room temperature. At the optimum cooling rate of 50 K/s, the nanorods had a diameter and length range of approximately 560 nm and 2~11 μm, respectively, with the highest saturation magnetization of 7.5 emu/g and a maximum coercivity of 120 Oe. Additionally, the nanorods exhibited good oxidation resistance with an antioxidation temperature of 883 K. These properties make the fabricated Mn_5_Si_3_ nanorods potentially useful for magnetic storage applications.

## Figures and Tables

**Table 1:** Nominal chemical compositions of brass alloys with different contents of Mn and Si (wt.%).

Brass Alloy	Zn	Mn	Si	Al	Cu
B-0.1Mn-0.03Si	35.0	0.10	0.03	3.0	bal.
B-0.2Mn-0.06Si	35.0	0.20	0.06	3.0	bal.
B-0.33Mn-0.1Si	35.0	0.33	0.10	3.0	bal.

**Table 2:** Summary of nanostructured Mn_5_Si_3_ prepared via different methods.

Sample	Preparation Method	Nanostructure	Dimension		M*s* (emu/g)	H*c* (Oe)
			Diameter	Length		
Ref. [15]	CVD	Nanorods	200–400 nm	Several μm	4.63	61.5
Ref. [14]	CVD	Nanorods	~1.82 μm	~43.92 μm	0.70	100
This work	Casting-extraction	Nanorods	400–750 nm	2~11 μm	7.5	120
Ref. [12]	Magnetron sputtering	Nanoparticles	~8.6 nm	-	~129	~500

## Data Availability

The data that support the findings of this study are available from the corresponding author, Hang Li, upon reasonable request.
